# Novel colored hydroxypropyl methyl cellulose/ magnetite carbon dots films for beef packaging with DFT calculations and molecular docking study

**DOI:** 10.1038/s41598-025-92976-9

**Published:** 2025-03-25

**Authors:** Hebat-Allah S. Tohamy

**Affiliations:** Cellulose and Paper Department, National Research Centre, 33 El Bohouth Str, P.O. 12622, Dokki Giza, Egypt

**Keywords:** Colored sensors, Magnetite carbon Dots, Food packaging, Biosensors, Bacterial detection, pH-sensor, Sulfur, Chemistry, Materials science, Nanoscience and technology

## Abstract

This study investigates the preparation and characterization of a novel HPMC-MCDs (Hydroxypropyl methylcellulose-magnetite carbon dots) composite film for potential applications in food safety monitoring. While carbon dots (CDs) offer promising sensing capabilities, their inherent lack of color limits direct visual detection, a limitation addressed in this work by incorporating magnetite (Fe_3_O_4_) to create a visually discernible sensor. Characterization techniques, including XRD, FTIR, and SEM, confirmed the successful integration of MCDs within the HPMC matrix. The incorporation of MCDs significantly reduced the film’s surface roughness. The HPMC-MCDs composite exhibited a remarkably smooth surface. DFT calculations revealed enhanced stability of the HPMC-MCDs composite. Fluorescence studies demonstrated color change in the HPMC-MCDs upon interaction with ***Salmonella enterica*** and ***B. cereus***, suggesting potential for bacterial detection. Furthermore, the HPMC-MCDs film exhibited pH-sensitive behavior, changing color in response to pH variations, making it a promising candidate for visual monitoring of beef meat spoilage. These findings suggest that HPMC-MCDs have the potential to serve as a multifunctional sensing platform for food safety applications.

Hydroxypropyl methylcellulose (HPMC) is a versatile cellulose ether widely used in food packaging^1–3^. HPMC emerges as a sustainable alternative to traditional petroleum-based food packaging^4^. It forms edible films and coatings that extend the shelf life of food products by acting as a protective barrier. These films reduce moisture loss and oxygen transmission, preventing spoilage and maintaining freshness^5–7^. Furthermore, HPMC films can be customized with additives like antimicrobial agents to enhance food safety and protect against light degradation. This versatility makes HPMC a valuable tool in modern food packaging, contributing to both food preservation and consumer safety^8–10^.

Food spoilage, triggered by harmful bacteria, poses a serious threat to public health^11–13^. Consuming spoiled food can result in a variety of foodborne illnesses, ranging from mild symptoms to severe, life-threatening conditions. To minimize these risks, early detection of food spoilage bacteria is essential^14–17^. By identifying and discarding contaminated food, we can effectively prevent the spread of illness and safeguard public health. Furthermore, early detection helps maintain the quality and integrity of food products. Spoiled food often exhibits undesirable qualities like off odors, unpleasant tastes, and altered textures, which can significantly diminish consumer satisfaction^18,19^. Various methods are utilized to detect food spoilage bacteria, including traditional culture techniques, rapid methods, and sensory evaluation^20–22^. By employing these methods, food safety experts and consumers can work together to ensure the safety and quality of the food supply.

Magnetite carbon dots (MCDs) offer a promising alternative to traditional carbon dots (CDs) for developing advanced food packaging sensors^23–27^. While CDs exhibit strong fluorescence properties, making them suitable for detecting various food quality parameters, MCDs possess additional magnetic properties that enhance their functionality^28–30^. These magnetic properties enable easier manipulation, improved stability, and the potential for multiple sensing modes^31,32^. By leveraging the advantages of MCDs, researchers can create innovative food packaging solutions that improve food safety, extend shelf life, and enhance consumer confidence. Microwave-assisted synthesis provides a swift and environmentally friendly method for producing MCDs, surpassing traditional techniques like co-precipitation. This approach drastically reduces reaction time and avoids the use of harmful chemicals commonly employed in co-precipitation. By exposing a blend of carbon precursors and iron salts to microwave radiation, MCDs can be synthesized in mere minutes, leading to a more efficient and sustainable process. This streamlined method not only expedites production but also minimizes environmental harm, making microwave-assisted synthesis a promising avenue for the large-scale production of MCDs for diverse applications, including food packaging sensors. Utilizing agricultural waste as a carbon source for MCD synthesis presents a sustainable and eco-friendly solution. By repurposing these abundant and often discarded materials, we can reduce environmental pollution and foster circular economy principles^29^. Improper management of agricultural waste can lead to various environmental issues, including soil degradation, water pollution, and greenhouse gas emissions^31–36^. Transforming these waste materials into valuable products like MCDs not only mitigates their negative impacts but also opens doors for sustainable development and innovation^29,37^.

During storage, meat proteins undergo a breakdown process, resulting in the formation of amino acids and peptides. This breakdown leads to an increase in the total volatile basic nitrogen (total-N) content. Consequently, total-N has been widely recognized as a crucial parameter for assessing meat quality^38,39^. *Salmonella* and *Staphylococcus aureus* are two major bacterial contaminants that can compromise food safety in beef^40–42^. *Salmonella* can cause foodborne illness, manifesting as symptoms like diarrhea, abdominal cramps, and fever. It can enter the beef supply chain at various stages, including animal husbandry, processing, and improper handling^43,44^. *Staphylococcus aureus*, on the other hand, produces toxins that lead to staphylococcal food poisoning, characterized by nausea, vomiting, and diarrhea^45,46^. Poor hygiene during food preparation and handling can facilitate its presence in beef^47^. To mitigate the risk of foodborne illness, it is essential to adhere to proper food safety practices, such as sourcing beef from reliable suppliers, storing it correctly, cooking it to a safe internal temperature, maintaining good hygiene, and preventing cross-contamination^48,49^.

Despite advancements in food packaging, challenges remain in developing multifunctional materials that are also scalable for widespread application. Current sensor technologies often lack the sensitivity and visual cues needed for rapid, on-site assessment of food quality. By harnessing the potential of HPMC and advanced technologies like MCD-based sensors, the future of food packaging is promising. Through continued research and development, we can create a future where food is not only safe and nutritious but also preserved efficiently, reducing waste and ensuring food security for generations to come. By harnessing the potential of HPMC and advanced technologies like MCD-based sensors, the future of food packaging is promising. Through continued research and development, we can create a future where food is not only safe and nutritious but also preserved efficiently, reducing waste and ensuring food security for generations to come.

## Experiment

### Materials

The sugarcane bagasse (SB) was obtained from the Paper Industry Quena Company, Egypt, and used to prepare N–CDs. Ferrous and ferric chloride (FeCl_3_ and FeCl_2_) were purchased from Sigma-Aldrich. Also, hydroxypropyl methyl cellulose (HPMC) and urea were purchased from the same place.

### Preparation of nitrogen-doped carbon Dots (N-CDs)

A mixture of 30 mg SB, 70 mg sodium hydroxide, 2400 mg urea, and 100 ml water was stirred (100 rpm at room temperature) for 30 min to form a homogeneous solution. This solution was frozen overnight, thawed, and then sonicated for 2 min to break up clumps. Finally, it was microwaved at 700 watts for 7 min^16^.

### Preparation of magnetite/nitrogen-doped carbon quantum Dots (MCDs)

MCDs were synthesized using microwave irradiation with FeCl_3_ and FeCl_2_ as precursors. N–CDs were dispersed in water and sonicated, then mixed with the iron precursor solution. The pH was adjusted to 11–12 with NaOH, and the mixture was microwaved. The resulting MCDs were magnetically separated, washed, and dried^29^.

### Hydroxypropyl methyl cellulose/ magnetite-nitrogen-doped carbon dots films

To prepare HPMC-MCDs film, 1 gm of HPMC was dispersed in 50 ml water and stirred at 50 °C until it was entirely solved. Then the HPMC solution was mixed with 1 gm MCDs with continuous stirring for 15 min. Then the mixture was poured inside Teflon plate and kept at 60 °C until the film formation without stirring. This film was denoted as HPMC-MCDs. The same formulation was prepared without MCDs for comparison and denoted as HPMC.

### Hydroxypropyl methyl cellulose/ magnetite-nitrogen-doped carbon dots films to monitor and preserve the beef

The fresh beef meat was purchased from the local meat shop in Cairo, Egypt. To remove the adhered blood, the beef meat was rinsed with Milli-Q water and subsequently air-dried in a laminar air flow hood. Beef was employed throughout this study and all samples were purchased at a local market. The samples were cut into portions weighing 10 g. The six beef samples were packed in a petri dish and stored at 4 °C for 21 days. During this time, one beef sample was randomly selected and tested on the 1st, 4th, 8th, 11th, 15th, and 21th days of storage^50^.

### Characterization

Scanning electron microscopy (SEM) images were taken using a Quanta/250-FEG scanning electron microscope (Thermo Fisher Scientific, Waltham, MA, USA). Fluorescence microscopy was performed using a Jasco FP-6500 spectrofluorometer (made in Japan) with a 150-watt xenon arc lamp. The FTIR spectra were taken with a Mattson 5000 spectrometer (Unicam, United Kingdom) using the potassium bromide (KBr) disk method. The mean hydrogen bond strength (MHBS) and the empirical crystallinity (LOI) were calculated as follows^51–53^:

1 $$\:\text{M}\text{H}\text{B}\text{S}=\frac{\text{A}\text{O}\text{H}\:}{\text{A}\text{C}\text{H}}$$

2 $$\:\text{L}\text{O}\text{I}=\frac{\text{A}1425\:}{\text{A}900}$$

Density functional theory (DFT) calculations were performed using the Gaussian 09 W program with the B3LYP/6-31G(d) level of theory.

3 $$\:{E}_{gap}=({E}_{LUMO}-{E}_{HOMO})$$

4 $$\:{\upeta\:}=\frac{({E}_{LUMO}+\:{E}_{HOMO})\:\:}{2}\:$$

5 $$\:{\upsigma\:}=\frac{1\:\:}{{\upeta\:}}$$

6 $$\:\text{S}=\frac{1\:\:}{2{\upeta\:}}$$

7 $$\:{\upomega\:}=\frac{{\text{P}\text{i}}^{2}\:}{{\upeta\:}}$$

8 $$\:{\Delta\:}{N}_{max}=\frac{-\text{P}\text{i}\:\:}{{\upeta\:}}$$

Where total energy is E_T_, the energy of the highest occupied MO is E_HOMO_, the energy of the lowest unoccupied MO is E_LUMO_, the energy gap is E_g_, the dipole moment is µ, the absolute hardness is η, the absolute softness is σ, the chemical softness is S, the global electrophilicity is ω, and the additional electronic charge is ΔN_max_^29,54^. The molecular docking of HPMC-MCDs was fabricated using standard bond length, with the Gaussian 09 W and detected by discovery Studio Client (version 4.2).

## Results and discussion

### XRD of N-CDs and MCDs

**Fig. 1:**
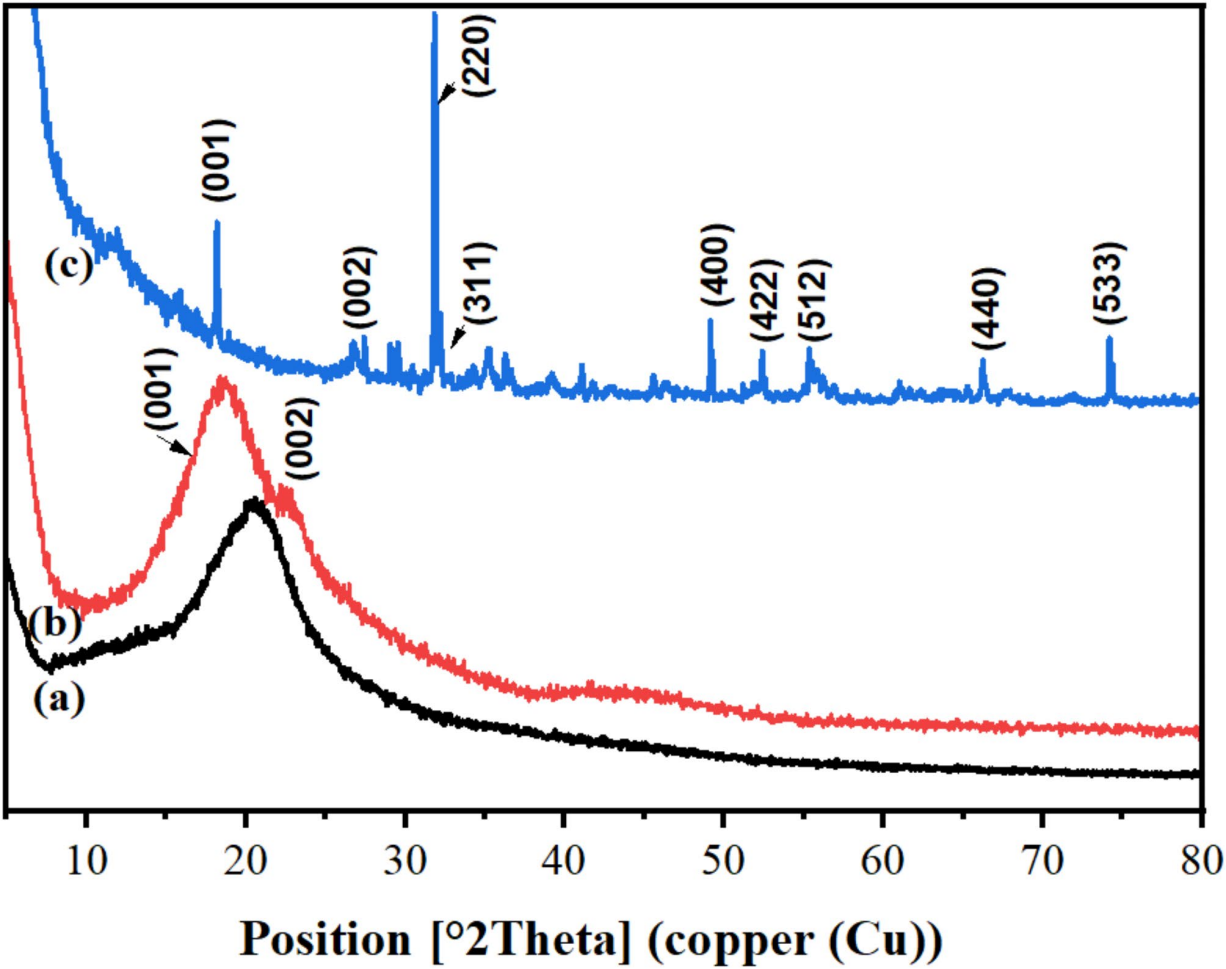
XRD of (**a**) HPMC, (**b**) N-CDs and (**c**) MCDs.

HPMC showed amorphous one broad peak around 20.28°, displaying the typical profile of HPMC. Figure 1 displays the X-ray diffraction patterns of N–CQDs and MCDs (i.e. Fe_3_O_4_/N–CQDs), which revealed peaks at 18.38 & 18.18 and 22.82 & 26.77° related to the (001) and (002) planes, respectively, due to the pesence of graphitc sheets with the d $\:\approx\:$ 3.81 and 3.32 A°^13,20^. The peaks at 31.81, 32.21, 49.14, 52.39, 55.34, 66.22, and 74.11° for MCDs are related to the (220), (311), (400), (422), (512), (440), and (533) crystal planes as evidence for the presence of Fe_3_O_4_ (JCPDS 01-076-7166)^29^. The d value of N–CQDs is higher than MCDs. This may be due to the presence of more Fe_3_O_4_ groups on N–CQDs which increase the number of oxygen groups. The calculated crystallinity for HPMC, N–CQDs and MCDs was 32.75, 44.53 and 21.15%.

### DFT calculations

**Fig. 2:**
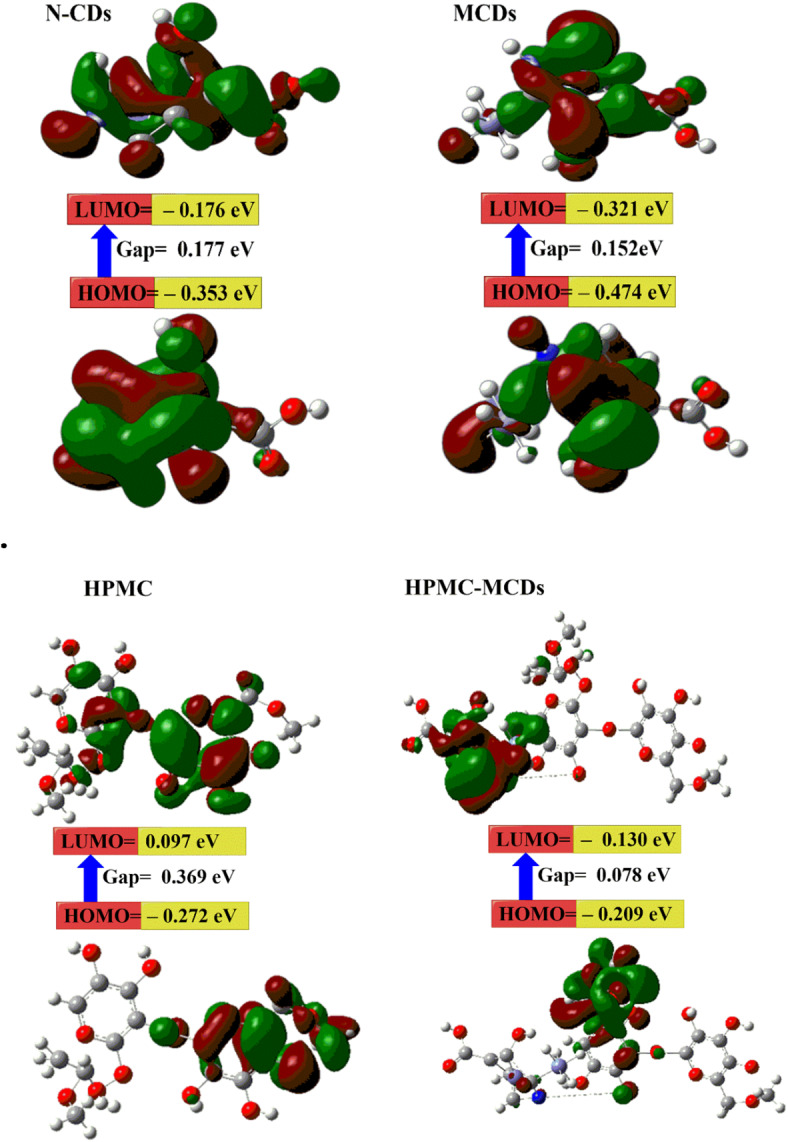
The gap energies (HOMO–LUMO) (eV) were calculated by using DFT B3LYP/6–31G (d), as was the molecular orbital interaction between N-CDs, MCDs, HPMC, and HPMC-MCDs.

DFT calculations were used to investigate the stability of N-CDs, MCDs, HPMC, and HPMC-MCDs. As shown in Fig. 2; Table 1, the results indicate that:

- The E_T_ of HPMC-MCDs (– 4485.50 au) is lower than HPMC (– 1466.76 au), N-CDs (– 2768.06 au) and MCDs (– 3026.52 au) which means that the HPMC-MCDs is more stable because of the chemical reaction between MCDs and HPMC^17,51^.
- The µ for HPMC-MCDs (i.e. 27.00 Debye) is higher than HPMC (i.e. 24.98 Debye). In addition, the electrophilicity for HPMC-MCDs (i.e. 0.3670 eV) is higher than HPMC (i.e. 0.0207 eV). This may be because of the presence of more Fe_3_O_4_, N and O atoms from MCDs which increase electronegativity in HPMC-MCDs compared to HPMC^29,51^.
- The calculated E_g_ for HPMC-MCDs is the lowest (i.e. 00.078 eV) compared to HPMC (i.e. 0.369 eV) due to the strong chemical reaction between HPMC and MCDs in the HPMC-MCDs^16,17^.
- The HPMC-MCDs is much softer (i.e. 12.706 eV) than the HPMC film (i.e. 2.704 eV) which is a good approximation of the strong energy changes between the donor (HOMO) and acceptor (LUMO) in HPMC-MCDs^29,54^.

**Table 1:** The quantum chemical parameters of N-CDs, MCDs, HPMC and HPMC-MCDs.

DFT B3LYP/6–31G (d)	*N*-CDs	MCDs	HPMC	HPMC-MCDs
E_LUMO_ (eV)	– 0.176	– 0.321	0.097	– 0.130
E_HOMO_(eV)	– 0.353	– 0.474	– 0.272	– 0.209
E_g_(eV)	0.177	0.152	0.369	0.078
E_T_(au)	– 2768.06	– 3026.52	– 1466.76	– 4485.50
μ (Debye)	– 3.934	6.354	24.98	27.00
ɳ (eV)	0.088	0.076	0.184	0.039
σ (eV)	11.299	13.118	5.408	25.412
S (eV)	5.649	6.559	2.704	12.706
Ω	0.3952	1.0379	0.0207	0.3670

### Fourier transform infrared spectroscopy (FTIR) spectra

**Fig. 3:**
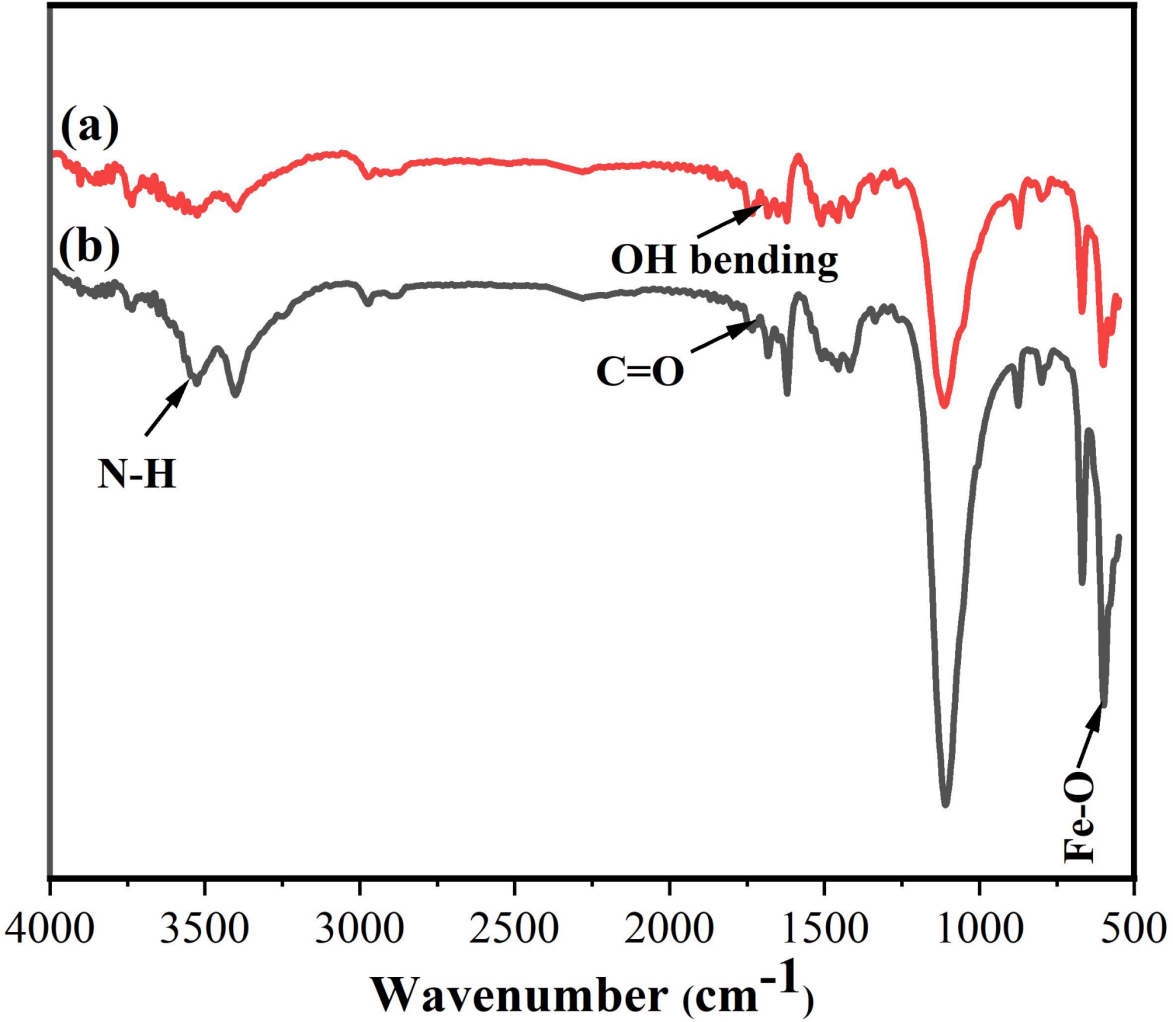
FTIR spectra of (**a**) HPMC, and (**b**) HPMC-MCDs.

Figure 3 illustrates the FTIR which was used to evaluate the structural changes of HPMC before and after MCDs incorporation. The FTIR spectrum of HPMC film shows strong absorption bands at the wavenumbers of 3525, 2975, 1509, 1455, 1118, and 873 cm^−1^, related to the stretching vibrations of the O–H, C–H, C–O bending, C–H bending, C–O–C pyranose ring vibration, β-glycosidic linkage (Fig. 3a). The low intensity band at 1718 cm^−1^ is related to the –OH bending vibration of water molecules^51^. Compared to the FTIR of HPMC film, the FTIR of HPMC-MCDs show additional, peak at 3544, 1733, and 601 cm^−1^ (Fig. 3b), which are due to the presence of N–H, C = O, Fe–O as a result of the incorporation of MCDs^29^. The HPMC-MCDs showed O–H band which shifted to lower wavenumber (i.e. 3405 cm^−1^) is a prove of more extensive hydrogen bonding networks. This was also proved from the calculated MHBS (Table 2). In addition, the LOI of HPMC-MCDs (i.e. 1.77) is higher the HPMC (1.23) which meaning more crystallinity and bonding due to the incorporation of Fe_3_O_4_ within HPMC in HPMC-MCDs^53,55,56^.

**Table 2:** The MHBS and LOI of HPMC and HPMC-MCDs.

Sample	HPMC	HPMC-MCDs
MHBS	0.90	0.91
LOI	1.23	1.77

### Morphological observations

**Fig. 4:**
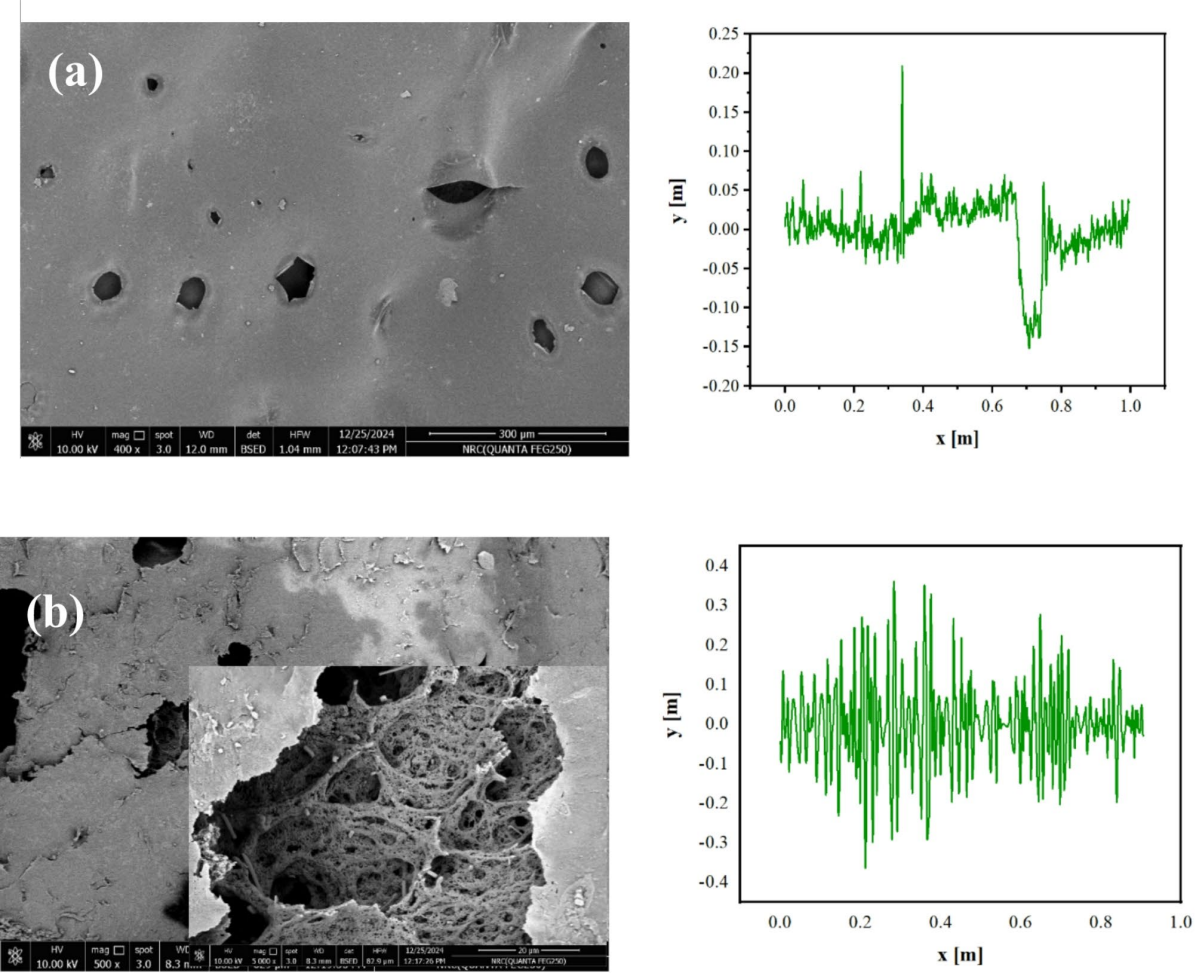
SEM analysis and roughness plot for (**a**) HPMC and (**b**) HPMC-MCDs.

Figure 4 illustrates the surface morphology of the HPMC, and HPMC-MCDs films. The HPMC showed a porous shaped film with pore size ranged between 40.78 and 5.61 μm, while, HPMC-MCDs showed a porous with honey combed pores due to the presence of MCDs. The porosity for HPMC-MCDs is equal to 20.65–27.57 μm. The observed high honeycombed porosity within the HPMC-MCDs sensory films offers several advantages. Porous structures facilitate enhanced interaction between the MCDs sensor and the surrounding environment. This increased accessibility leads to improved sensitivity and faster response times from the sensor.

The addition of MCDs to HPMC dramatically reduced the surface roughness of the resulting HPMC-MCDs composite film. The HPMC film exhibited a roughness average (Ra) of 10.7585 and a root mean square roughness (Rq) of 16.2462. Upon incorporation of MCDs, these values plummeted to an Ra of 0.072497 and Rq of 0.100751. This significant decrease in roughness indicates a smoother surface morphology in the HPMC-MCDs composite compared to the HPMC film alone. The smoother surface suggests that the MCDs have a notable impact on the film’s microstructure, potentially filling voids or creating a more uniform and ordered arrangement within the HPMC matrix. This change in surface properties could influence various applications of the film, such as its interaction with other materials, its optical properties, or its performance in sensing or catalytic processes.

### Fluorescent microscope and Molecular docking

**Fig. 5:**
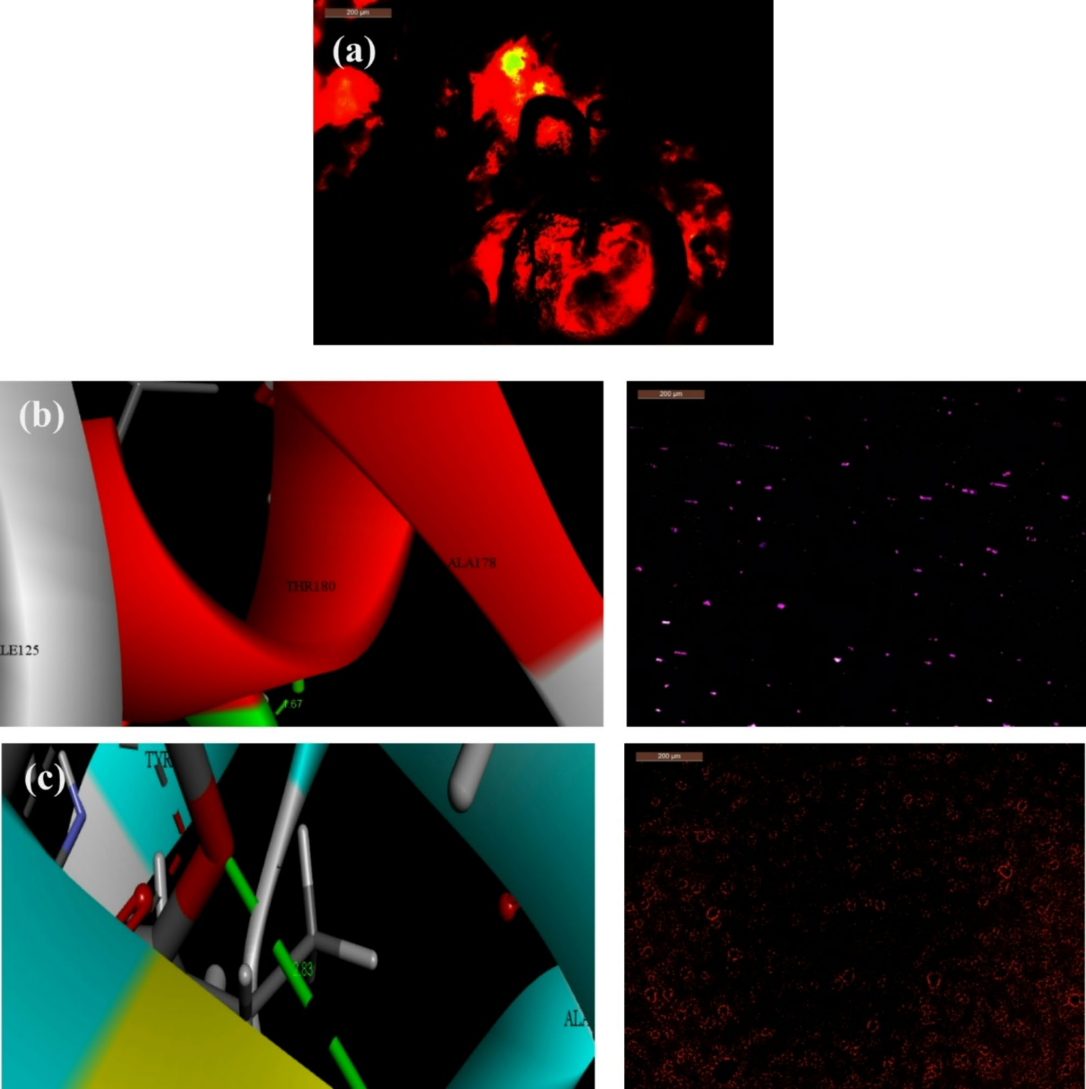
(**a**) Fluorescent microscope for HPMC-MCDs, (**b**) Fluorescent microscope and molecular docking of HPMC-MCDs as a ligand against *Salmonella enterica* PDB (6CH3), and (**c**) Fluorescent microscope and molecular docking of HPMC-MCDs as a ligand against *B. cereus* PDB (2K5Q).

The HPMC-MCDs showed intense red/orange fluorescence which proved their fluorescent properties (Fig. 5a). After HPMC-MCDs contact with *Salmonela* and *B. cereus* the fluorescence structure and color changed to small spotted purple shapes in HPMC-MCDs/ *Salmonela* and a round red shapes have a perforate structure in HPMC-MCDs/ *B. cereus*. Figure 5 illustrated the biological activity of HPMC-MCDs as a ligand against *Salmonella enterica* PDB (6CH3) and *B. cereus* PDB (2K5Q) as receptors. The HPMC-MCDs showed binding with *Salmonella enterica* and *B. cereus* protein with bond length ~ 1.67 and 2.83 A°, respectively (Fig. 5b&c), which means the high binding between HPMC-MCDs and *Salmonella enterica*. This will be proved after by fluorescent microscope which will show a change in the color tone of HPMC-MCDs from red/orange to purple. As a Gram-negative bacterium, *Salmonella* possesses an outer membrane rich in lipopolysaccharide (LPS). This membrane serves as a crucial barrier, shielding the bacterium from environmental stressors and contributing to its virulence. LPS molecules are embedded within this membrane, extending outwards to form a protective layer. The presence of LPS may interfere with the fluorescence properties of MCDs, resulting in a shift in the emitted light color to purple. The observed change from intense red/orange fluorescence (Fig. 5a) to small spotted purple shapes upon *Salmonella* exposure suggests a potential interaction between the HPMC-MCDs and bacterial LPS (Fig. 5b)On the other hand, *B. cereu*s, as a Gram-positive bacterium, possesses teichoic acids in its cell wall, which carry a negative charge. The negative charge of *B. cereus*’s surface may facilitate the electrostatic attraction and binding of positively charged molecules which result in the structure changing from the intense red/orange before contacting with bacteriaFig. . 5a) to red structure with cavities upon *B. cereu*s exposure Fig. 5c)^16,17^.

### HPMC-MCDs film as pH-sensor for beef meat spoilage by naked eye

**Fig. 6:**
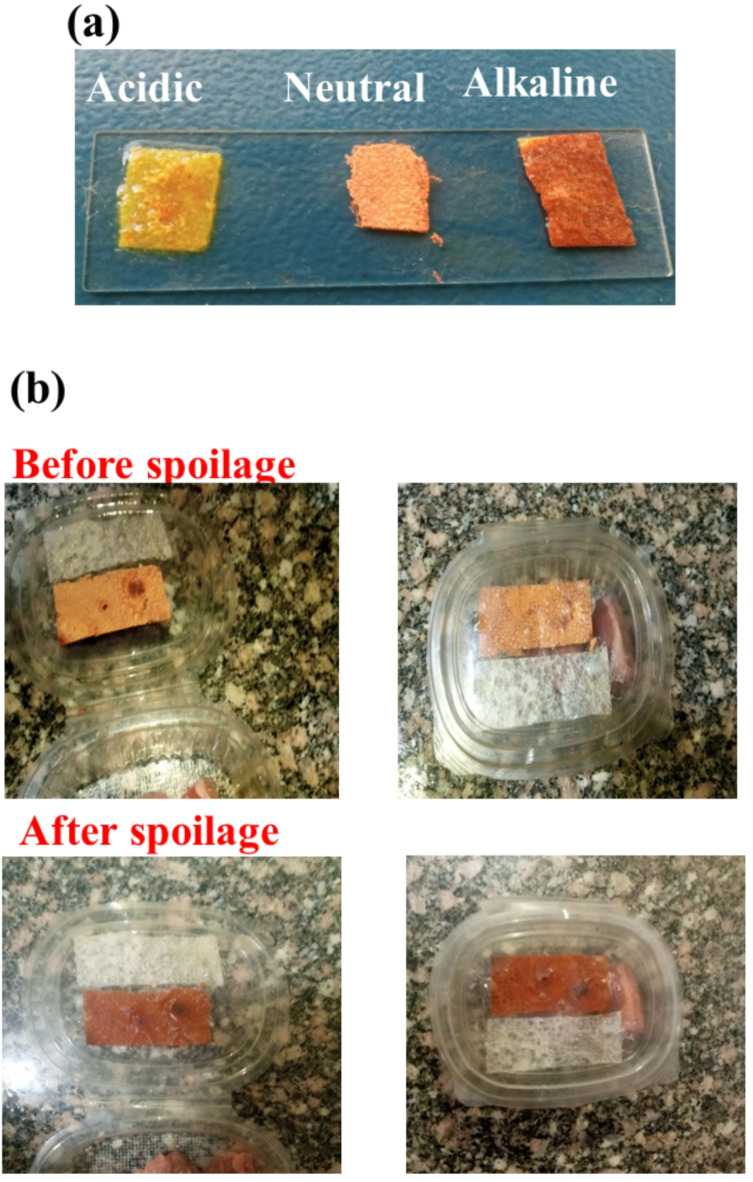
(**a**) Color response of HPMC-MCDs film at acidic, neutral and alkaline media, (**b**) Testing of HPMC and HPMC-MCDs films on beef meat spoilage,

Beef typically exhibits a pH within a narrow range of 5.4 to 5.7 ^57^. Bacterial activity during spoilage can lead to an elevation in pH as alkaline compounds are produced^58,59^. While *Salmonella* itself doesn’t directly cause a significant pH increase, its presence within spoiled meat can contribute to an elevated pH. As *Salmonella* and other spoilage bacteria proliferate, they produce various metabolic byproducts, some of which are alkaline in nature. The accumulation of these alkaline byproducts can gradually raise the overall pH of the meat^60,61^. To assess pH sensitivity, we conducted a test on the HPMC-MCDs film. It exhibited a color change – yellow under acidic conditions, orange under neutral and red under alkaline conditions (Fig. 6a). This visual response eliminates the need for specialized equipment. The clear color change, easily observable with the naked eye, provides a simple and convenient method for monitoring pH levels. Significantly, the color response of the HPMC-MCDs film is directly influenced by beef meat spoilage, resulting in a pronounced color change. When compared to pure HPMC film, the developed HPMC-MCDs film (Fig. 6b) exhibits a distinct color transition from yellow to red over time, demonstrating its sensitivity to beef meat spoilage.

## Conclusions

This study successfully synthesized and characterized HPMC-MCDs, demonstrating their potential as a novel sensing platform. XRD analysis confirmed the successful incorporation of Fe_3_O_4_ nanoparticles within the N-CDs. DFT calculations revealed that HPMC-MCDs exhibit enhanced stability, higher electronegativity, and a reduced band gap compared to individual components. FTIR spectroscopy confirmed the successful integration of MCDs within the HPMC matrix. Morphological analysis revealed a porous structure in HPMC-MCDs, potentially enhancing sensor sensitivity. Fluorescence studies demonstrated a distinct color change in HPMC-MCDs upon interaction with *Salmonella* and *B. cereus*, suggesting potential for bacterial detection. This was further supported by molecular docking studies, which showed strong binding affinities of HPMC-MCDs with *Salmonella enterica* and *B. cereus* proteins. Finally, the HPMC-MCDs film exhibited pH-sensitive behavior, changing color from yellow to red in response to increasing pH. This pH sensitivity, coupled with the observed bacterial-induced fluorescence changes, demonstrates the potential of HPMC-MCDs as a simple and effective visual indicator for beef meat spoilage. Several avenues for future research emerge from these findings. A key area of exploration is enhancing the sensor’s specificity and sensitivity for target bacterial strains, particularly in complex real-world food matrices. This could involve investigating different surface modifications or incorporating selective binding agents. Furthermore, a crucial next step is to correlate the observed pH-sensitive color changes with established indicators of food spoilage, such as total volatile basic nitrogen or microbial counts, to validate its effectiveness as a practical freshness indicator. From a materials perspective, exploring different CDs precursors or synthesis methods could potentially enhance the sensing performance. Finally, the potential of this platform could be expanded by investigating its applicability to other food products beyond beef and exploring the incorporation of additional sensing modalities, such as temperature or gas detection, for a more comprehensive food quality assessment tool.

## Data Availability

Data is provided within the manuscript.

## References

[1] Yin, Y. & Woo, M. W. Transitioning of petroleum-based plastic food packaging to sustainable bio-based alternatives. *Sustainable Food Technol.* **2**, 548–566 (2024).

[2] Keldibekova, R., Suleimenova, S., Nurgozhina, G. & Kopishev, E. *Interpolymer Complexes Based Cellulose Ethers: Application Polym.* **15**, 3326 (2023). 10.3390/polym15153326 PMC10422396 37571220

[3] Mottola, S., Viscusi, G., Tohamy, H. A. S., El-Sakhawy, M. & Gorrasi, G. Marco, I. Application of electrospun N-doped carbon Dots loaded cellulose acetate membranes as cationic dyes adsorbent. *J. Environ. Manage.* **370**, 122714 (2024). De. 39383756 10.1016/j.jenvman.2024.122714

[4] Romão, S., Bettencourt, A. & Ribeiro, I. A. Novel features of cellulose-based films as sustainable alternatives for food packaging. *Polymers* **14**, 4968 (2022). 36433095 10.3390/polym14224968 PMC9699531

[5] Díaz-Montes, E. & Castro-Muñoz, R. Edible films and coatings as food-quality preservers: an overview. *Foods* **10**, 249 (2021). 33530469 10.3390/foods10020249 PMC7912451

[6] Kocira, A. et al. Polysaccharides as edible films and coatings: characteristics and influence on fruit and vegetable quality—A review. *Agronomy* **11**, 813 (2021).

[7] Shahidi, F. & Hossain, A. Preservation of aquatic food using edible films and coatings containing essential oils: A review. *Crit. Rev. Food Sci. Nutr.* **62**, 66–105 (2022). 32847401 10.1080/10408398.2020.1812048

[8] Ghadermazi, R., Hamdipour, S., Sadeghi, K. & Ghadermazi, R. Khosrowshahi Asl, A. Effect of various additives on the properties of the films and coatings derived from hydroxypropyl methylcellulose—A review. *Food Sci. Nutr.* **7**, 3363–3377 (2019). 31762990 10.1002/fsn3.1206 PMC6848826

[9] Sebti, I., Chollet, E., Degraeve, P., Noel, C. & Peyrol, E. Water sensitivity, antimicrobial, and physicochemical analyses of edible films based on HPMC and/or Chitosan. *J. Agric. Food Chem.* **55**, 693–699 (2007). 17263462 10.1021/jf062013n

[10] Vasile, C. & Baican, M. Progresses in food packaging, food quality, and safety—controlled-release antioxidant and/or antimicrobial packaging. *Molecules* **26**, 1263 (2021). 33652755 10.3390/molecules26051263 PMC7956554

[11] Ibrahim, S. A. et al. Lactic acid bacteria as antimicrobial agents: food safety and microbial food spoilage prevention. *Foods* **10**, 3131 (2021). 34945682 10.3390/foods10123131 PMC8701396

[12] Karanth, S., Feng, S., Patra, D. & Pradhan, A. K. Linking microbial contamination to food spoilage and food waste: the role of smart packaging, spoilage risk assessments, and date labeling. *Front. Microbiol.* **14**, 1198124 (2023). 37426008 10.3389/fmicb.2023.1198124 PMC10325786

[13] Misiou, O. & Koutsoumanis, K. Climate change and its implications for food safety and spoilage. *Trends Food Sci. Technol.* **126**, 142–152 (2022).

[14] Akinsemolu, A. A. & Onyeaka, H. N. *In Food Safety and Quality in the Global South 489–531* (Springer, 2024).

[15] Gourama, H. *In Food Safety Engineering 25–49* (Springer, 2020).

[16] Tohamy, H. A. S. & Novel Speedy, and Eco-Friendly carboxymethyl Cellulose-Nitrogen doped carbon Dots biosensors with DFT calculations, molecular docking, and experimental validation. *Gels* **10**, 686 (2024). 39590042 10.3390/gels10110686 PMC11593792

[17] Tohamy, H. A. S. Cellulosic schiff base hydrogel biosensor for bacterial detection with pH/thermo-responsitivity: DFT calculations and molecular Docking. *Int. J. Biol. Macromol.* **283**, 137389 (2024). 39537077 10.1016/j.ijbiomac.2024.137389

[18] Dodero, A., Escher, A., Bertucci, S., Castellano, M. & Lova, P. Intelligent packaging for real-time monitoring of food-quality: current and future developments. *Appl. Sci.* **11**, 3532 (2021).

[19] Ling, E. K. & Wahab, S. N. Integrity of food supply chain: going beyond food safety and food quality. *Int. J. Productivity Qual. Manage.* **29**, 216–232 (2020).

[20] Ferone, M., Gowen, A., Fanning, S. & Scannell, A. G. Microbial detection and identification methods: bench top assays to omics approaches. *Compr. Rev. Food Sci. Food Saf.* **19**, 3106–3129 (2020). 33337061 10.1111/1541-4337.12618

[21] Kumar, A., Kulshreshtha, S., Shrivastava, A. & Saini, A. Biosensors for food spoilage detection: a comprehensive review of current advances. *J. Food Chem. Nanotechnol*. **10**, S73–S82 (2024).

[22] Vasavada, P. C., Lee, A. & Betts, R. Conventional and novel rapid methods for detection and enumeration of microorganisms. *Food Saf. Eng.*, 85–128 (2020).

[23] Kayani, K. F., Ghafoor, D., Mohammed, S. J. & Shatery, O. B. Carbon Dots: synthesis, sensing mechanisms, and potential applications as promising materials for glucose sensors. *Nanoscale Adv.* (2024). 10.1039/d4na00763h PMC11583430 39583130

[24] Nejad, F. G., Tajik, S., Beitollahi, H. & Sheikhshoaie, I. Magnetic nanomaterials based electrochemical (bio) sensors for food analysis. *Talanta* **228**, 122075 (2021). 33773704 10.1016/j.talanta.2020.122075

[25] Wang, H., Jiang, S., Xu, Z. & Xu, L. A novel fluorescent sensor based on a magnetic covalent organic framework-supported, carbon dot-embedded molecularly imprinted composite for the specific optosensing of bisphenol A in foods. *Sens. Actuators B*. **361**, 131729 (2022).

[26] Eissa, M. S. et al. Magnetic molecularly imprinted polymers and carbon Dots molecularly imprinted polymers for green micro-extraction and analysis of pharmaceuticals in a variety of matrices. *Microchem. J.*, 111235 (2024).

[27] Tohamy, H. A. S., El-Sakhawy, M. & Kamel, S. A. Greener future: carbon nanomaterials from lignocellulose. *J. Renew. Mater.* **13** (2025).

[28] Strachota, B. et al. Potential environmental application of a tough and Temperature-Responsive nanocomposite hydrogel based on Poly (N-Isopropylacrylamide-co-Sodium Methacrylate) and clay. *Int. J. Environ. Res.* **19**, 7 (2025).

[29] Tohamy, H. A. S. Speedy synthesis of magnetite/carbon Dots for efficient chromium removal and reduction: a combined experimental and DFT approach. *Emergent Mater.*, 1–13 (2024).

[30] Tohamy, H. A. S. et al. Development and characterization of fluorescent cellulose succinate hydrogels for efficient chromium adsorption. *J. Polym. Res.* **31**, 339 (2024).

[31] Khan, M. A., Sun, J., Li, B., Przybysz, A. & Kosel, J. Magnetic sensors-A review and recent technologies. *Eng. Res. Express*. **3**, 022005 (2021).

[32] Wang, S. et al. Magnetic nanostructures: rational design and fabrication strategies toward diverse applications. *Chem. Rev.* **122**, 5411–5475 (2022). 35014799 10.1021/acs.chemrev.1c00370

[33] Abdel-Fatah, A. S. et al. Anatase-cellulose acetate for reinforced desalination membrane with antibacterial properties. *BMC Chem.* **17**, 112 (2023). 37700386 10.1186/s13065-023-01013-1 PMC10496367

[34] Al Kiey, S. A. & Tohamy, H. A. S. Sustainable energy harvesting: manganese oxide-decorated carbon quantum Dots derived from agriculture for high-performance supercapacitors. *J. Energy Storage*. **101**, 113758 (2024).

[35] El-Sakhawy, M., Tohamy, H. A. S., AbdelMohsen, M. M. & El-Missiry, M. Biodegradable carboxymethyl cellulose based material for sustainable/active food packaging application. *J. Thermoplast. Compos. Mater.* **37**, 2035–2050 (2024).

[36] Tohamy, H. A. S., El-Sakhawy, M., Abdel-Halim, S. A., El-Masry, H. M. & AbdelMohsen, M. M. Antimicrobial Plectranthus amboinicus emulsions prepared with amphiphilic cellulose stearate. *Euro-Mediterranean J. Environ. Integr.*, 1–12 (2024).

[37] Tohamy, H. A. S., Mohamed, F., E.-Z., S. & El-Sakhawy, M. Novel microwave assisted carboxymethyl-graphene oxide and its hepatoprotective activity. *BMC Pharmacol. Toxicol.* **25**, 50 (2024). 39138519 10.1186/s40360-024-00768-0 PMC11321068

[38] Lee, H., Kim, M. S., Lee, W. H. & Cho, B. K. Determination of the total volatile basic nitrogen (TVB-N) content in pork meat using hyperspectral fluorescence imaging. *Sens. Actuators B*. **259**, 532–539 (2018).

[39] Xiong, L., Hu, Y., Liu, C. & Chen, K. Detection of total volatile basic nitrogen (TVB-N) in pork using fourier transform near-infrared (FT-NIR) spectroscopy and cluster analysis for quality assurance. *Trans. ASABE*. **55**, 2245–2250 (2012).

[40] Adesiji, Y. O., Alli, O. T., Adekanle, M. A. & Jolayemi, J. B. Prevalence of Arcobacter, Escherichia coli, Staphylococcus aureus and Salmonella species in retail Raw chicken, pork, beef and goat meat in Osogbo, Nigeria. *Sierra Leone J. Biomedical Res.* **3**, 8–12 (2011).

[41] Kadariya, J., Smith, T. C. & Thapaliya, D. Staphylococcus aureus and staphylococcal food-borne disease: an ongoing challenge in public health. *BioMed research international* 827965 (2014). (2014). 10.1155/2014/827965 PMC3988705 24804250

[42] Odo, S. E., Uchechukwu, C. F. & Ezemadu, U. R. Foodborne diseases and intoxication in Nigeria: prevalence of Escherichia coli 0157: H7, Salmonella, Shigella and Staphylococcus aureus. *J. Adv. Microbiol.* **20**, 84–94 (2021).

[43] Al-Seghayer, M. S. & Al-Sarraj, F. The outbreak of foodborne disease by pathogenic Enterobacteriaceae antimicrobial resistance-a review. *Asian Food Sci. J.* **20**, 91–99 (2021).

[44] Mahamedin, M. M., Mahamedin, M. M. & Ed-Dra, A. *In Microbial Toxins in Food Systems: Causes, Mechanisms, Complications, and Metabolism*277–288 (Springer, 2024).

[45] Abdallah, E. M. & Sulieman, A. M. E. Staphylococcus aureus. Microbial Toxins in Food Systems: Causes, Mechanisms, Complications, and Metabolism, 235 (2024).

[46] Narayan, K. G., Sinha, D. K. & Singh, D. K. *In Veterinary Public Health & Epidemiology: Veterinary Public Health-Epidemiology-Zoonosis-One Health*301–308 (Springer, 2023).

[47] Gutema, F. D. et al. Assessment of hygienic practices in beef cattle slaughterhouses and retail shops in Bishoftu, Ethiopia: implications for public health. *Int. J. Environ. Res. Public Health*. **18**, 2729 (2021). 33800319 10.3390/ijerph18052729 PMC7967449

[48] Mahunu, G., Osei-Kwarteng, M., Ogwu, M. C. & Afoakwah, N. A. *In Food Safety and Quality in the Global South 427–461* (Springer, 2024).

[49] Nabwiire, L. *Assessing Beef Vendors’ Compliance With Food Safety Standards in Kamuli District, Uganda, and Consumers’ Beef Handling Practices in the US Virgin Islands* (Iowa State University, 2023).

[50] Yu, K. et al. Stretchable, antifatigue, and intelligent nanocellulose hydrogel colorimetric film for real-time visual detection of beef freshness. *Int. J. Biol. Macromol.* **268**, 131602 (2024). 38626836 10.1016/j.ijbiomac.2024.131602

[51] Tohamy, H. A. S., Greener, S. & Packaging Carbon Nanotubes/Gelatin-Enhanced Recycled Paper for Fire Retardation with DFT Calculations. Journal of Renewable Materials, {pages}.

[52] El-Sakhawy, M., Abdel-Halim, S. A., Tohamy, H. A. S., El-Masry, H. M. & AbdelMohsen, M. M. Amphiphilic Carboxymethyl Cellulose Stearate for Pickering Emulsions and Antimicrobial Activity of Chrysanthemum Essential Oil. (2025).

[53] Hassanein, H. D., Tohamy, H. A. S., AbdelMohsen, M. M., El-Masry, H. M. & El-Sakhawy, M. Preparation and characterization of cellulose acetate/corn silk extract films for potential antimicrobial application. *Egypt. J. Chem.* (2024).

[54] Tohamy, H. A. S. Carboxymethyl hemicellulose hydrogel as a fluorescent biosensor for bacterial and fungal detection with DFT and molecular Docking studies. *Sci. Rep.* **15**, 741 (2025). 39753654 10.1038/s41598-024-83157-1 PMC11699063

[55] Viscusi, G., Mottola, S., Tohamy, HA.S., Gorrasi, G., De Marco, I. Design of Cellulose Acetate Electrospun Membranes Loaded with N-doped Carbon Quantum Dots for Water Remediation. In: Mannina, G., Ng, H.Y. (eds) Frontiers in Membrane Technology. IWA-RMTC 2024. Lecture Notes in Civil Engineering, vol 525. Springer, Cham. 10.1007/978-3-031-63357-7_22 (2024).

[56] EL-Nashar, D., Tohamy, H. A. & Koriem, A. Improving acrylonitrile rubber properties by recycled nano graphene oxide prepared from sugarcane Bagasse. *Polym. Compos.* 1–11. 10.1002/pc.29578 (2025).

[57] Young, O., Zhang, S., Farouk, M. & Podmore, C. Effects of pH adjustment with phosphates on attributes and functionalities of normal and high pH beef. *Meat Sci.* **70**, 133–139 (2005). 22063289 10.1016/j.meatsci.2004.12.018

[58] Ledenbach, L. H. & Marshall, R. T. Microbiological spoilage of dairy products. *Compendium Microbiol. Spoilage Foods Beverages*, 41–67 (2009).

[59] Dilbaghi, N. & Sharma, S. Food spoilage, food infections and intoxications caused by microorganisms and methods for their detection Hisar-125001. (2007).

[60] Deminicis, R. G. D. S. & Deminicis, B. B. Microbiological and pH of Ground Beef Sold in Supermarkets in Southern Bahia: Caracterização Microbiológica E pH De Carne Bovina Moída Comercializada Em Supermercados No Sul Da Bahia. Scientia Agraria Paranaensis, 431–436 .

[61] Rood, L. *Shelf-life Extension, Spoilage Community, High Spoilage Potential, Meat pH* (University of Tasmania, 2022).

